# Correlated Resting-State Functional MRI Activity of Frontostriatal, Thalamic, Temporal, and Cerebellar Brain Regions Differentiates Stroke Survivors with High Compared to Low Depressive Symptom Scores

**DOI:** 10.1155/2019/2357107

**Published:** 2019-07-28

**Authors:** Peter Goodin, Gemma Lamp, Rishma Vidyasagar, Alan Connelly, Stephen Rose, Bruce C. V. Campbell, Tamara Tse, Henry Ma, David Howells, Graeme J. Hankey, Stephen Davis, Geoffrey Donnan, Leeanne M. Carey

**Affiliations:** ^1^Neurorehabilitation and Recovery, Stroke, Florey Institute of Neuroscience and Mental Health, Melbourne Brain Centre-Austin Campus, Heidelberg, Victoria, Australia; ^2^Department of Medicine and Neurology, Melbourne Brain Centre, Royal Melbourne Hospital, Parkville, Melbourne, Victoria, Australia; ^3^Advanced Imaging, Florey Institute of Neuroscience and Mental Health, Melbourne Brain Centre-Austin Campus, Heidelberg, Victoria, Australia; ^4^Australian e-Health Research Centre, CSIRO, Brisbane, Queensland, Australia; ^5^Occupational Therapy, School of Allied Health, Human Services and Sport, College of Science, Health and Engineering, La Trobe University, Bundoora, Victoria, Australia; ^6^Department of Medicine, School of Clinical Sciences, Monash University, Clayton, Victoria, Australia; ^7^Department of Florey, University of Melbourne, Parkville, Victoria, Australia; ^8^School of Medicine, University of Tasmania, Australia; ^9^Medical School, University of Western Australia, Perth, Western Australia, Australia

## Abstract

**Background:**

One in three survivors of stroke experience poststroke depression (PSD). PSD has been linked with poorer recovery of function and cognition, yet our understanding of potential mechanisms is currently limited. Alterations in resting-state functional MRI have been investigated to a limited extent. Fluctuations in low frequency signal are reported, but it is unknown if interactions are present between the level of depressive symptom score and intrinsic brain activity in varying brain regions.

**Objective:**

To investigate potential interaction effects between whole-brain resting-state activity and depressive symptoms in stroke survivors with low and high levels of depressive symptoms.

**Methods:**

A cross-sectional analysis of 63 stroke survivors who were assessed at 3 months poststroke for depression, using the Montgomery–Åsberg Depression Rating Scale (MÅDRS-SIGMA), and for brain activity using fMRI. A MÅDRS-SIGMA score of >8 was classified as high depressive symptoms. Fractional amplitude of frequency fluctuations (fALFF) data across three frequency bands (broadband, i.e., ~0.01–0.08; subbands, i.e., slow-5: ~0.01–0.027 Hz, slow-4: 0.027–0.07) was examined.

**Results:**

Of the 63 stroke survivors, 38 were classified as “low-depressive symptoms” and 25 as “high depressive symptoms.” Six had a past history of depression. We found interaction effects across frequency bands in several brain regions that differentiated the two groups. The broadband analysis revealed interaction effects in the left insula and the left superior temporal lobe. The subband analysis showed contrasting fALFF response between the two groups in the left thalamus, right caudate, and left cerebellum. Across the three frequency bands, we found contrasting fALFF response in areas within the fronto-limbic-thalamic network and cerebellum.

**Conclusions:**

We provide evidence that fALFF is sensitive to changes in poststroke depressive symptom severity and implicates frontostriatal and cerebellar regions, consistent with previous studies. The use of multiband analysis could be an effective method to examine neural correlates of depression after stroke. The START-PrePARE trial is registered with the Australian New Zealand Clinical Trial Registry, number ACTRN12610000987066.

## 1. Introduction

Post stroke, patients frequently experience motor, sensory, cognitive, and behavioural changes, all of which may impact recovery [1]. Changes to a stroke survivor's mood are also common [2], with depression as the most frequently reported psychiatric disorder following ischaemic stroke [3]. Poststroke depression (PSD) is estimated to affect approximately one-third of survivors [4, 5], compared to about one-sixth of the nonstroke population [6, 7].

PSD is associated with poorer recovery prospects [8], including increased disability [9], worse cognitive outcomes [10–12], decreased quality of life [13], and increased risk of mortality [14]. In particular, PSD negatively impacts response to rehabilitation in acute and subacute phases of recovery [15]. However, our understanding of the potential mechanisms underlying the negative impact of depressive symptoms on recovery and rehabilitation is currently limited. Determining factors that may assist in the identification of those “at risk” of developing poststroke depression may aide in the recovery process and/or prediction of response to rehabilitation.

The value of biomarkers of stroke recovery that focus on brain structure and function has recently been highlighted in consensus-based recommendations [16]. Neuroimaging markers of depression may be used to provide new insight into neural mechanisms underlying depression, to predict the likelihood of future depressive symptoms, and/or to predict readiness to engage in treatment or treatment response. All are important reasons to identify stroke survivors with underlying vulnerabilities that may be “at risk” of developing depression.

One approach has been to investigate the relationship between lesion location and depression; however, despite a large number of studies, findings are equivocal [17–20]. These findings suggest that lesion location alone is unlikely to be an informative biomarker associated with PSD. A meta-analysis of behavioural, biochemical, and neuroimaging markers of PSD found associations with reduced cerebral blood flow and regional volume reductions [21].

In the broader literature of clinical depression, the disorder is not considered to be caused by independent, localised changes within specific brain regions but is thought to be partially due to disruption of communication between areas [22]. Several meta-analyses of fMRI cohort studies of clinical depression have found changes in brain activation and connectivity [23–25]. Findings highlight alteration of brain regions consistent with the current system-level models of depression. It may therefore be useful to examine biomarkers of PSD using resting-state methods that focus on intrinsic brain activity and whole brain [26].

Resting-state fMRI methods focus on low frequency fluctuations (LFF) present within the blood oxygen level-dependent (BOLD) signal (0.01 to ~0.1 Hz) [27] which in part reflect intrinsic neuronal activity [28, 29]. Several methods have been developed that evaluate different aspects of the signal. For example, local or regional correlations between BOLD time series are able to be examined, collectively known as functional connectivity [30]. These functional connectivity analyses focus on temporal correlations of the BOLD signal.

The spectral (frequency) characteristics of signal within individual voxels during resting-state can also be examined, typically by taking the sum amplitude of low frequency fluctuations (ALFF) [31] or a ratio of LFF over the entire estimated spectra (fractional ALFF, fALFF) [32]. Of these two methods, fALFF has been shown to be robust against physiological artefacts and vascular effects [33, 34], which are common poststroke given changes to neurovasculature post-stroke [35].

While methods typically focus on the full LFF range, spectral measures allow the exploration of subbands, which have been suggested to be important for a scope of physiological and function processes within the brain [36, 37]. Wang et al. [38, 39] used fALFF to examine LFF and subbands of slow-5 (0.01–0.027 Hz) and slow-4 (0.027–0.07) in medication of naive participants with major depressive disorder over two studies. Both studies found similar changes in LFF measures when depressed participants were compared to controls.

Wang et al. [39] also found areas that displayed an interaction effect between controls and those with depression and subband signal changes. Their results showed that the areas of the left ventromedial prefrontal cortex, left inferior frontal gyrus, and bilateral precuneus showed changes in amplitude in the slow-5 band, but not slow-4. This suggests that examination of subbands may be useful in identifying regions that are associated with depressive symptoms. It also highlights the value of investigating for an interaction effect in brain regions.

To date, PSD studies of resting-state changes have not been widely employed, have focused on functional connectivity from specific regions, e.g., within the default mode network (DMN) and anterior cingulate, and have included participants of varying times post stroke. Results from these studies have been inconsistent. For example, Lassalle-Lagadec et al. [40] found correlations at 10 days post stroke between the depression score and the left middle temporal cortex and precuneus and at 3 months with the neostriatum. Vicentini et al. [41] found an association with the posterior cingulate cortex and depression score at approximately 1-month poststroke, while Liu et al. [42] failed to find any regional correlations of the posterior cingulate with a depression score in a cohort of chronic stroke survivors. More recently, Balaev et al. [43] explored changes in the default mode network and found changes post treatment. Only one study, by Egorova et al. [44], used voxelwise spectral analysis of fALFF and found mean differences between depressed and nondepressed stroke survivors in the frontal and insular regions.

While examining main effects that can be informative for identifying brain regions for further examination, they give no information regarding how intrinsic activity in these regions may influence the individual depressive symptom score. Furthermore, finding regions that show differing response depending on regional activity may help identify potential biomarkers and predict severity of depressive symptoms [45, 46].

Our aim was to examine the interaction effects between the amplitude of whole-brain resting-state signal (using broadband and subband fALFF) and depressive symptom score, in stroke survivors with high and low levels of depressive symptoms. We hypothesised that there would be a significant interaction effect in frontolimbic regions with the depressive symptom score, such that the high depressive score group would show a positive association between the depressive symptom score and increases in regional brain signal response.

## 2. Methods

### 2.1. Participants

Sixty-three participants (44 female) with advanced imaging from the STroke imAging pRevention and Treatment (START) cohort [47] were included in the current study. Participants were three months post their first ischaemic stroke episode, diagnosed clinically, and confirmed via brain imaging. They were required to be medically stable, be 18 years or older, speak English, not have a significant disability prior to stroke, and be able to give informed consent. Stroke participants were excluded if they had a brainstem infarct, previous neurological dysfunction, or evidence of unilateral spatial neglect or were not suitable for MRI.

### 2.2. Clinical Data, Depression Symptom Assessment, and Group Formation

Data was obtained on age, sex, and history of depression prior to their stroke. Stroke severity was measured using the National Institute of Health Stroke Scale (NIHSS) [48]. We assessed for depressive symptoms using the structured interview guide for the Montgomery–Åsberg Depression Rating Scale (MÅDRS-SIGMA) [49]. The MÅDRS-SIGMA is a 10-item structured interview that enquires into participants' range of depressive symptoms including reported sadness, inner tension, concentration difficulties, and pessimistic thoughts. Each item is scored on a range from 0 (no symptoms present) to 6 (high levels of symptoms present). Total scores on the MÅDRS-SIGMA range from 0 to 60 with higher scores indicating higher levels of depressive symptoms. Participants were placed into low or high depressive symptom groups based on the MÅDRS-SIGMA cut-off score of >8. This cut-off was chosen as it has been shown to give the optimal sensitivity and specificity (0.85, 0.71, AUC = 0.91 [95%CI = 0.84–0.98]) for the classification of poststroke depression from a sample of 150 stroke patients [50].

### 2.3. MRI Acquisition

Imaging data was acquired on a 3 Tesla Siemens Trio scanner. Resting-state functional data was acquired using an echo planar imaging (EPI) sequence over 7 minutes (TR = 3000 ms, TE = 30 ms, 3 mm isotropic voxels, 72 × 72 matrix, 44 slices, 216 mm FOV). Participants were instructed to “Close your eyes and rest. You do not need to think about anything in particular” and were also instructed that they should stay awake throughout the scan. Following the scan, this was confirmed by participant report. Resting-state acquisition was consistently conducted after a touch activation task to the fingertips.

A high-resolution 1 mm isotropic MPRAGE scan (TR = 1900 ms, TE = 2.55 ms, 256 × 256 matrix, 160 slices, 216 mm FOV) was collected for coregistration to the functional data, segmentation, and normalisation to MNI space. 2D FLAIR (fluid attenuation inverse recovery sequence; 1 mm isotropic, TR = 6000 ms, TE = 388 ms, 100 mm FOV) was acquired axially for delineation of infarcts.

### 2.4. Lesion Mask Creation

Axial FLAIR images were used to identify and draw a mask around the primary infarct hyperintensity using MRicron (http://www.mccauslandcenter.sc.edu/mricro/mricron/index.html). Masks were quality checked and modified as necessary by a neurologist (BC) to ensure they accurately represented the infarct.

### 2.5. Resting-State Preprocessing and Analysis

A customised data cleaning pipeline optimised for preprocessing of stroke data was constructed [51]. The pipeline used functions from DCMstack (https://github.com/moloney/dcmstack), Analysis of Functional NeuroImages (AFNI) [52], SPM12 v6685 (http://www.fil.ion.ucl.ac.uk/spm/software/spm12/), Advanced Normalization Tools (ANTs) [53], Numpy [54], Scipy [55], and Nibabel (https://github.com/nipy/nibabel), combined under the NiPype framework [56].

Anatomical image preprocessing consisted of segmentation using the new segmentation method and coregistration to the mean EPI image [51]. White matter and cerebrospinal fluid (CSF) masks were created by thresholding the segmented white matter and CSF images at 0.99 and eroding two times using a 3 × 3 × 3 mm structure element to minimise partial volume effects. Normalisation to Montreal Neurological Institute (MNI) space was achieved by transforming an MNI space 3 × 3 × 3 mm template image to subject space, then using the inverse transformation matrix to warp the T1 image from subject space to MNI space. Stroke participants had their FLAIR and lesion mask included in the pipeline, which were coregistered to the T1 image and coregistered to the EPI image [51].

Prior to preprocessing, we conducted systematic, visual quality inspection of each participant's resting-state data. Participants were excluded if their data were shown to have consistent, excessive motion or noticeable distortions. No participants were excluded on this basis. Preprocessing of EPI data included despiking, slice timing correction to the central slice, and realignment to the first volume. Motion and physiological related artefact were regressed from the data using the Friston 24 parameter model [57] and aCompCor [58], taking the top five components each for white matter and CSF mask extracted signals. The global signal from within the brain mask was also regressed. This can help attenuate residual motion and physiological effects not removed by prior cleaning [59]. In connectivity-based measures, global signal regression is thought to alter the covariance structure of the data, introducing artefactual negative correlations [60, 61]. However, as fALFF is derived from voxel-based spectral data, there is no evidence that this step impacts individual or group measures. After cleaning, images were normalised to MNI space using the inverse transform matrix computed from the EPI space T1 image. The data were then smoothed using a 6 mm Gaussian kernel.

### 2.6. fALFF Calculation and Analysis

We employed fALFF broadband and subband (slow-5/slow-4) measures to examine for potential associations between the resting-state brain activity and poststroke depressive symptom score. fALFF maps were calculated using the method outlined by Zuo et al. [34]. Briefly, data were linearly detrended and using Fast Fourier Transform, converted to the frequency domain. The square root of the transform was used to convert the power spectra to spectral magnitude. fALFF was defined as the voxel-wise ratio of the sum of LFF data (~0.01 Hz to 0.08 Hz) over the sum of the entire spectra (~0 Hz to 0.33 Hz). Slow-5 and slow-4 bands were calculated by taking frequency bands from ~0.01 to 0.027 and 0.027 to 0.07 Hz, respectively, and dividing over the entire spectra. Participant fALFF maps were then *z*-scored by subtracting the global fALFF mean value from each voxel and divided by the global fALFF standard deviation.

Second level analysis was performed in SPM 12 using cluster-based familywise error correction (cFWE) to control multiple comparisons, with the cluster forming threshold set to *p* < 0.001 [62] and spatial threshold set to *p* < 0.05.

An interaction model of the group × MÅDRS-SIGMA was used, which initially included covariates of age, sex, and NIHSS. No significant voxels were found for covariates (all cFWE > 0.05), so these were removed from the model and the data were reanalysed. Significant clusters were localised using the automatic anatomical labelling (AAL) atlas [63] as found in the Wake Forest University PickAtlas v3.0.5 [64, 65]. fALFF amplitudes for significant clusters were extracted and used in further analysis.

### 2.7. Statistical Analysis

Demographic data were analysed using the statistical package R [66] for between-group comparisons. We examined differences in sex membership and prestroke history of depression between groups using chi-square tests with *p* values simulated based on 10,000 reshuffles. Two sample *t*-tests were used to examine differences between groups for age and NIHSS.

### 2.8. Data Visualisation

fMRI data were visualised using mricroGL (http://www.mccauslandcenter.sc.edu/mricrogl/). Extracted fALFF data were visualised using Seaborn (https://seaborn.pydata.org/).

## 3. Results

### 3.1. Demographics

Demographic and clinical information for the low and high depressive symptom score groups is presented in Table 1.

Two sample *t*-tests showed no significant difference between the two groups for age (*t*(55.06) = 1.64, *p* = 0.10) or NIHSS (*t*(49.01) = 0.81, *p* = 0.42). Chi-square tests showed no significant difference between the groups for sex (*χ*^2^ = 0.67, *p* = 0.57).

The high depressive symptom score group showed a significantly increased MÅDRS-SIGMA score compared to the low group (*t*(27.83) = 9.08, *p* < 0.001), as expected. There were also significantly higher counts of reported sadness (*χ*^2^ = 5.99, *p* = 0.021), discouragement (*χ*^2^ = 7.58, *p* = 0.012,) and loss of interest in daily activities (*χ*^2^ = 7.72, *p* = 0.008) in the high depressive symptom score group compared to low. No significant differences were observed between the low and high groups for counts of antidepressant usage (*χ*^2^ = 2.23, *p* = 0.29).

The high depressive symptom score group showed a greater number of participants with a prestroke history of depression (*χ*^2^ = 10.08, *p* = 0.002). There did not appear to be a significant difference in the MÅDRS-SIGMA score between those with a history of depression (*M* = 20.00, SD = 8.53) and those without (*M* = 13.26, SD = 5.25) in the subgroup analysis of the high depressive symptom score group (*t*(6.25) = 1.83, *p* = 0.11).

### 3.2. Lesion Overlap

**Figure 1:**
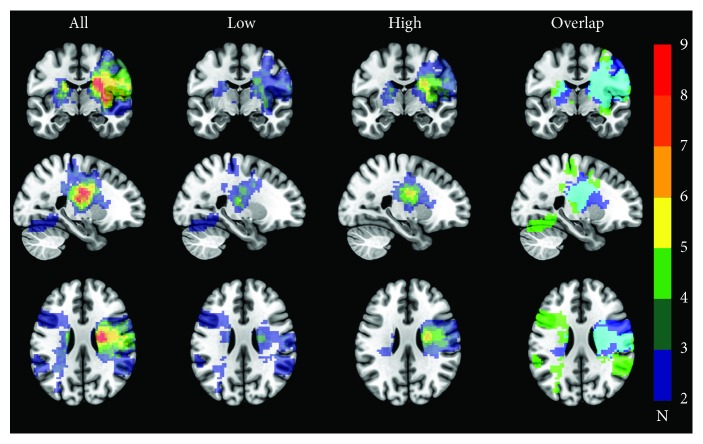
Overlap of lesion locations for all participants, low depressive symptom score group, high depressive symptom score group, and overlap of lesion location for the low and high groups. For columns All, Low, and High, cooler colours indicate lower numbers of participants with overlapping lesions, and warmer colours indicate higher numbers of participants with overlapping lesions. For the Overlap column, green = low depressive symptom score group, dark blue = high depressive symptom score group, and light blue = overlap between the two groups. All brain images are shown in neurological convention.

The overlap of lesion locations across all participants and the groups is shown in Figure 1. Lesion locations across all participants showed the largest overlap in the left and right hemispheres, in an area including the internal capsule, corona radiata, and insula. Damage to the right hemisphere extended to lateral parietal regions. The low and high depressive symptom score groups showed the greatest overlap in the right internal capsule/corona radiata.

### 3.3. Functional Connectivity fALFF Results

**Figure 2:**
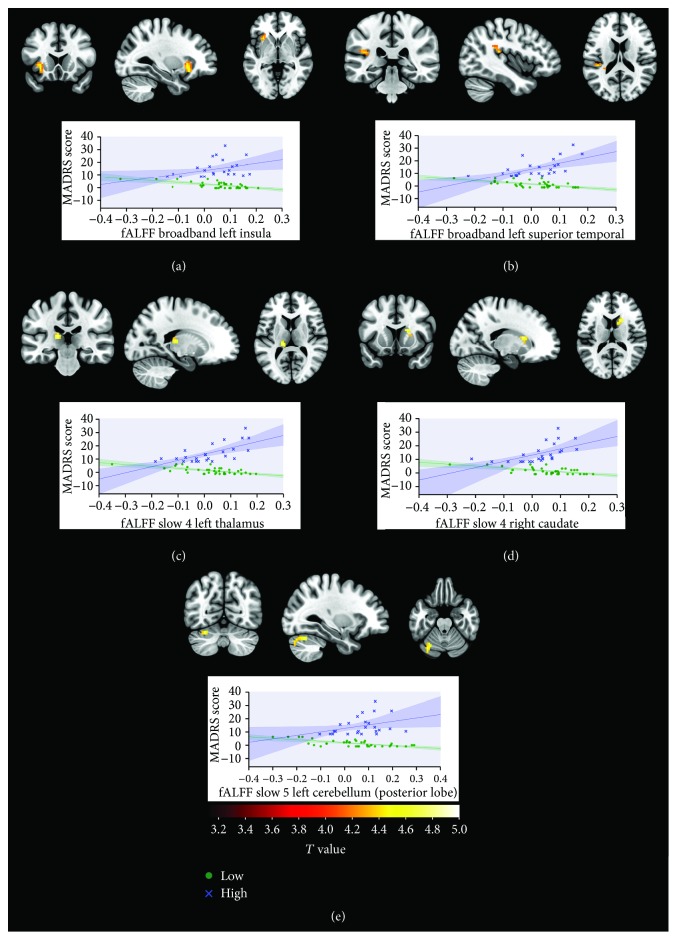
Cluster locations showing a significant interaction effect between the low and high depressive symptom score groups with the mean cluster fALFF response plotted against the MÅDRS-SIGMA score for broadband, slow-4 band, and slow-5 band (green represents low depressive symptom score group, blue represents high depressive symptom score group, and bands along regression line represent the 95% confidence interval). (a) Broadband: left insula. (b) Broadband: left superior temporal. (c) Slow-4: left thalamus. (d) Slow-4: right caudate. (e) Slow-5: left cerebellum. In the high depressive symptom group, high response from these regions was associated with an increased depressive symptom score. The low depressive symptom group showed no significant association between these regions and depressive symptom score. All brain images are shown in neurological convention.

Examination of the interaction between the group and MÅDRS-SIGMA score showed several regions across the three bands of interest that had an increased slope for the high depressive symptom score group compared to the low depressive symptom score group. No significant increases or decreases in the slope were found for the low depression symptom score group. Cluster location, coordinates, and summary information for the three bands are shown in Table 2. *R*-squared values were calculated using the formula *r*^2^ = √*t*^2^/(*t*^2^ + *df*), where *t* is the peak voxel value of the cluster and *df* is the degrees of freedom. Regions and cluster interaction effects are presented in Figure 2.

## 4. Discussion

A significant *interaction effect* was observed between groups with low and high depressive symptoms. Our data showed that for the high depressive symptom score group, (those who scored greater than 8 on the MÅDRS-SIGMA), the increased symptom score was associated with increased fALFF amplitude in the left insula, superior temporal lobe, thalamus and cerebellum, and right caudate. Conversely, no significant association was found between the fALFF amplitude and low depressive symptom score group. Such an interaction effect has not been previously described.

The interaction effect found adds a novel insight as it maps a linear relationship with signal changes in brain regions with depressive symptom scores, separable by low and high depressive symptoms. In addition, the fact that these differential effects were observed in the same regions for patients with and without depressive symptoms provides further support for a role for this set of regions in depression. For example, we observed an interaction effect in the insula. The insula has been extensively associated with depression in prior studies of nonstroke depression, yet it is unclear if insula is hyper- or hypoactivated, with variable reports depending on the use of positive or negative stimuli, the stage (first episode vs. repeated), or severity (major vs. subthreshold) of depression [67].

Our findings provide insight into how brain signal is differentially associated with the depression score in those with and without depressive symptoms. Further, these results suggest that MÅDRS-SIGMA and fALFF analyses could potentially be used to identify individuals at risk of developing poststroke depression. This is important given the negative impact poststroke depression has been shown to have on the participation of everyday activities [68] and readiness to engage in the process of rehabilitation [8], which has also been shown to be negatively impacted [9–13]. Additionally, these results suggest that analysis using multiple fALFF frequency bands may be better than single band to study neural correlates of PSD.

In the nonstroke depression literature, alterations of resting-state activity to depressive symptoms have been well established [69] and may even allow for the exploration of different depressive subgroups [70]. Meta-analyses focusing on studies of resting-state changes in participants with depression have found associations with a large number of areas across the brain, including cortical, subcortical, and cerebellar locations, that show divergence of response from healthy controls [23–25].

Despite suggestions that resting-state methods may be a better approach to examine changes post stroke [71, 72], they have not been widely employed in PSD research. Most studies that have used functional connectivity have focused on specific regions of interest to investigate connectivity changes, e.g., from a default mode network and anterior cingulate [40–43, 73]. These studies showed inconsistent results, potentially due to differing times post stroke, methods used, and possible inclusion of artefact, a common issue with correlation-based methods [58] if not adequately controlled for. In our study, we utilised fALFF, which has been shown to be less susceptible to physiological artefact [34], performed a voxel-wise approach, and examined broad and subfrequency bands. We found significant differences in response to the depression score as a function of fALFF amplitude between those who presented with low depressive symptoms and those with high depressive symptoms.

The five significant clusters we found were located within the left insula, superior temporal lobe, thalamus, cerebellum, and right caudate. The insula, thalamus, and caudate are all part of the fronto-limbic-thalamic circuit [74], which is thought to be a major component in the neurocircuitry of depressive illness [22, 75–79]. The posterior superior temporal lobe and insula are also associated with social emotional processing [80], although the involvement of the superior temporal region with depression is currently not well understood [81]. The insula has extensive connections to frontolimbic areas and has previously been linked to aberrant emotional and interoceptive processing in depression [67]. The insula is also reported to have a role in homeostasis through the regulation of sympathetic and parasympathetic systems [82], in salience and selective attention, especially during challenging tasks [83], in motor learning [84] and in motor recovery from stroke [85]. The cerebellum has long been known for connections with the somatomotor cortex [86, 87], but recently, subdivisions within the cerebellum have been discovered which are functionally connected with a wide range of cortical functional networks [88]. A recent voxel-based lesion symptom mapping study [89] also found an association between the cerebellum and a measure of depressive symptoms, the geriatric depression scale. Interestingly, their results are also within a subdivision of the left superior cerebellum, which has been shown to have extensive functional connections to networks of the cerebrum including cognitive, emotional processing and salience networks [90].

Examination of fALFF amplitudes across several bands allowed us to uncover associations between regions and PSD that otherwise would have been hidden. Examining the frequency characteristics of signals is a widely used method in electromagnetic physiology research [91, 92], but has been slow to emerge within functional MRI analyses. Subbands within the low frequency resting-state range have previously been identified [36, 37], and the use of spectral methods such as fALFF has shown distinct spatial differences between them [34]. If broadband range alone had been examined, alterations within the caudate, thalamus, and cerebellum would not have been detected. This suggests that within the “resting-state range” of 0.01 to 0.08 Hz, different regions have unique oscillatory characteristics which are associated with depressive symptom severity. In the nonstroke depression literature, these areas are thought to be involved in cognitive and emotional modulation [93] and are considered to be central to the emotional dysregulation that is a hallmark of the condition [94–96].

The analysis performed in this study had similarities with that described by Egorova et al. [44]. Egorova et al. also investigated stroke survivors at 3 months post stroke, but used a different measure of depression (Patient Health Questionnaire-9), which had a lower proportion with depression (31%), and 40% of their depressed group (8/20) had a prior stroke. Only 2% (1/44) of the nondepressed group had a prior stroke, potentially impacting the findings. Egorova et al. reported mean differences between the low and high depressive symptom score groups in the dorsolateral prefrontal, precentral, and middle frontal regions. In contrast, we did not find any significant differences between the groups in frontal regions. Similar to our study, Egorova et al. found a significant association with the depression scores, in the left insula/superior temporal gyrus; however, in contrast, this was observed in the slow-4 subband and not the broadband as we reported. Further, our study found an interaction effect in both of these regions separately. Differences in the findings could be due to the difference in models used to examine the data. Egorova et al. examined only the group main effects, which in the General Linear Model framework compares the distance between slopes of the groups. In contrast, an interaction term examines differences in how the slopes interact with a third variable (in this case MÅDRS-SIGMA scores). We did not test group differences, as in the presence of a significant interaction effect interpretation of main effects may be misleading [97]. Thus, the studies asked different, but complementary, questions.

A caveat of our study is that the groups were determined by the cut-off score rather than a diagnosis made by a clinician. The cut-off score of >8 was used in this study to denote the high depressive symptom score. This cut-off, determined by Sagen et al. [50], had an AUC of 0.91 to correctly classify the depressed state of poststroke patients. However, it must be acknowledged that depression is a multifaceted disorder with idiosyncratic presentation of symptoms and a simple cut-off score may incorrectly classify some individuals as depressed when they are not depressed and vice versa. Optimally, determination of group placement requires examination from a clinician trained to identify and make a diagnosis of mood disorders. A second caveat of this study is the presence of overlap between voxels that showed a significant interaction effect and voxels that were affected by lesion damage. These regions of overlap however occurred in voxels where 3 or less participants showed damage. This would have affected less than 8% of the total low MÅDRS-SIGMA group or less than 12% of the total high MÅDRS-SIGMA group and only in a subset of voxels where a significant interaction was found.

A few methodological issues and their potential impact are highlighted. A scanning acquisition time of 7 minutes was selected, following pilot protocol testing, to optimize comfort for the patient while achieving adequate signal-to-noise and robust findings. While longer acquisition periods may be recommended for resting-state functional connectivity analyses [98, 99], voxelwise methods based on BOLD frequency spectrum have been shown to reach a stable state around 5 minutes [100]. The impact of head motion on BOLD fluctuations was minimized by the following: careful preparation and support positioning of the patient; real-time monitoring of motion, with repeating scan if excessive motion was evident; systematic, quality inspection of each participant's resting-state data prior to inclusion in data analysis; motion regression and global signal regression (see Methods); and standardization (*z*-scoring) of ALFF and fALFF based on evidence that it serves to decouple amplitudes from head motion [100]. We did not censor periods of high motion from the analysis, as missing data may introduce artefact into the spectra [100]. Finally, delay in the hemodynamic response function of the BOLD response is identified as an issue impacting functional connectivity data analysis following stroke [101]. A potential advantage of using a frequency spectra analysis (rather than time domain analysis) is that the hemodynamic lag is not such an issue in spectral methods of analysis.

## 5. Conclusion and Implications

In summary, this study provides evidence that fALFF, a measure of resting-state activity, is sensitive to changes in poststroke depressive symptom severity and implicates the frontostriatal and cerebellar regions consistent with previous studies. Significant interaction effects between the resting-state fALFF values and MÅDRS-SIGMA score were observed in the left insula, superior temporal lobe, thalamus, and cerebellum and in the right caudate across several frequency bands. These regions showed differing activity patterns when coupled with the MÅDRS-SIGMA score between those who scored low on the MÅDRS-SIGMA compared to those who scored high. These results may be useful in identifying “at risk” individuals for PSD and guide further exploration of brain regions and networks vulnerable to altered functioning in PSD. Identification of “at risk” individuals has clinical implications for planning clinical pathways and as a factor in the effectiveness of rehabilitation therapies due to impact on a patient's “readiness for change,” which further impacts on when therapeutic interventions should be targeted.

Evidence of an interaction effect is also of value in better understanding the neural mechanisms underlying poststroke depression, in particular the association with the amplitude of resting signals of certain brain regions in different groups of patients. The possibility of manipulating brain signal activity in key areas, such as insula, to differentially impact depressive symptoms in stroke patients is appealing. Given the role of the insula in multimodal sensory and cognitive emotional processing, and in learning, the potential to influence brain signal activity through such experiences suggests a worthwhile area of investigation.

## Figures and Tables

**Table 1:** Demographic and clinical information for the low and high depressive symptom score groups.

Group	Low	High
*n*	38	25
Age (mean, SD)	64.68 (13.56)	59.28 (12.26)
Sex (no. of females)	28	19
MÅDRS-SIGMA score (mean, SD)	2.29 (2.31)	14.88 (6.67)^∗∗∗^
History of depression	0/38 (0%)	6/25 (24%)^∗∗^
Reported sadness (yes/no)	5/33 (13.1%)	15/10^∗^ (60%)
Reported discouragement (yes/no)	4/34 (10.5%)	15/10^∗∗^ (60%)
Reported loss of interest (yes/no)	3/35 (7.9%)	16/9^∗∗^ (64%)
On antidepressant medication (yes/no)	1/37 (2.6%)	3/22 (13.6%)
NIHSS (mean, SD)	0.58 (1.20)	0.84 (1.28)

^∗^ *p* < 0.05, ^∗∗^*p* < 0.01, and ^∗∗∗^*p* < 0.001. MÅDRS-SIGMA = Montgomery–Åsberg Depression Rating Scale using Structured Interview Guide.

**Table 2:** Regions that showed significant group × MÅDRS-SIGMA score interaction effects for broadband (0.01–0.08 Hz), slow-4 band (0.027 to 0.067 Hz), and slow-5 band (0.01–0.027 Hz) of interest. Peak voxel region, coordinates in the MNI space, cluster size (*k*) and statistical values (*t*, *z*, *r*^2^, and *p*) of regions are reported.

Region	MNI coords (*xyz*)	*k*	*t*/*z*	*r* ^2^	*p*
*Broadband*					
Left superior temporal lobe	-36 -39 27	72	5.06/4.59	0.30	0.006
Left insula	-30 21 -9	49	4.82/4.41	0.28	0.035
*Slow-4*					
Left thalamus	-15 -27 12	44	4.60/4.24	0.26	0.025
Right caudate	18 12 15	59	4.48/4.14	0.25	0.006
*Slow-5*					
Left cerebellum, posterior lobe	-30 -66 -24	45	4.28/3.98	0.24	0.030

## Data Availability

The neuroimaging and clinical data used to support the findings of this study are restricted by the Human Research Ethics Committee of Austin Health, Australia, in order to protect patients' confidentiality. Data may be made available from Dr. Leeanne Carey, l.carey@latrobe.edu.au, for researchers who meet the criteria for access to this confidential data.
